# Brimonidine Eye Drops Causing Encephalopathy in a Patient With Advanced Chronic Kidney Disease

**DOI:** 10.7759/cureus.17725

**Published:** 2021-09-05

**Authors:** Benjamin Y.F. So, Kit Ming Lee, Arthur H.C. Tang, Terence Yip

**Affiliations:** 1 Medicine, Queen Mary Hospital, Hong Kong, HKG; 2 Medicine/Nephrology, Tung Wah Hospital, Hong Kong, HKG

**Keywords:** brimonidine, dialysis, chronic kidney disease, encephalopathy, eye drops

## Abstract

Brimonidine eye drops are frequently prescribed for the treatment of glaucoma and ocular hypertension in adults. Systemic toxicities including neurological side effects have been reported with its use, especially in the paediatric population. In this report, we present a case of encephalopathy secondary to the use of brimonidine eye drops in a patient with underlying advanced chronic kidney disease, who recovered promptly after drug cessation. Herein, we also review the pharmacokinetics of eye drops leading to their systemic side effects, especially in the context of renal impairment. We also explore the possibility of extracorporeal treatment, such as by haemodialysis, for the treatment of these manifestations. This case demonstrates the need to clarify a patient’s drug history and stop offending medications early on in a patient with delirium, while treatments such as antidotes or extracorporeal treatment are being considered.

## Introduction

Brimonidine, an α_2_-adrenergic agonist commonly prescribed for the treatment of glaucoma and ocular hypertension, is a lipid-soluble compound structurally related to clonidine. Systemic side effects have been reported after ocular administration of brimonidine, the most severe of which are neuropsychiatric manifestations [1]. While these cases seem more common in the paediatric population, there are only a few reports on brimonidine toxicity in adult patients. Here, we report on a case of acute encephalopathy attributed to the use of brimonidine tartrate eye drops in a 66-year-old woman with advanced chronic kidney disease (CKD). Her presentation was partly modified by the use of different modalities of renal replacement therapy (RRT) during her hospitalization. To the best of our knowledge, no reports have explored this clinical entity in the context of severe renal failure.

## Case presentation

A 66-year-old woman was admitted to the intensive care unit (ICU) for acute diverticulitis with septic shock. Her past medical history was significant for hypertension, diabetes mellitus, hyperlipidaemia, gout, glaucoma, and stage 5 CKD. One month before admission, her creatinine was 395 μmol/L (4.47 mg/dL), corresponding to an estimated glomerular filtration rate of 10 mL/min/1.73 m2. She was a housewife who was cognitively intact and completely independent in her activities of daily living before admission.

She was conservatively managed with intravenous antibiotics and vasopressors. She also developed acute kidney injury with oliguria. RRT was initiated in view of uraemia, metabolic acidosis, and hyperkalaemia, and the patient received a prolonged session of continuous venovenous haemofiltration (CVVH) during her ICU stay. Upon resolution of sepsis, her renal function had recovered to its previous baseline, and RRT was discontinued.

She was noted to be increasingly confused and agitated in the days following discharge from the ICU. Her Montreal Cognitive Assessment (Hong Kong version) (HK-MoCA) score was 3/30. Computed tomography scan and magnetic resonance imaging of the brain showed age-related cerebral atrophy. Lumbar puncture was performed and cerebrospinal fluid analysis was grossly unremarkable. Septic screens including blood and urine cultures were negative. Her blood tests are presented in Table 1. There was a mild leukocytosis that was already significantly downtrend from previous values. There were no electrolyte disturbances that could account for her altered mental state. Her renal function was similar to the previous baseline. Vitamin B12, folate, ammonia, red cell transketolase levels, and thyroid function tests, were all within the normal range. Electroencephalogram showed generalized symmetrical moderate background slowing with intermittent frontal delta activity. The findings were non-specific and merely reflected underlying encephalopathy. In view of these findings, regular intermittent haemodialysis twice a week was resumed on suspicion of uraemic encephalopathy, but no improvement was seen after two weeks. The patient remained frankly delirious and pulled out her haemodialysis catheter, requiring limb restraints.

**Table 1:** Blood results during an episode of delirium ALT: alanine aminotransferase; AST: aspartate aminotransferase.

Variable	Value	Reference range
Haemoglobin (g/dL)	8.8	11.5 – 14.8
White blood cell (x 10^9^/L)	13.75	3.89 – 9.93
Platelet (x 10^9^/L)	272	167 – 396
Sodium (mmol/L)	137	136 – 148
Potassium (mmol/L)	4.1	3.6 – 5.0
Chloride (mmol/L)	97	100 – 109
Bicarbonate (mmol/L)	28	21 – 31
Urea (mmol/L)	26.1	2.9 – 8.0
Creatinine (μmol/L)	363	49 – 82
Calcium (albumin-adjusted) (mmol/L)	2.51	2.24 – 2.63
Phosphate (mmol/L)	0.92	0.88 – 1.45
Albumin (g/L)	32	39 – 50
Globulin (g/L)	30	26 – 40
Bilirubin (μmol/L)	11	4 – 23
Alkaline phosphatase (U/L)	192	47 – 124
ALT (U/L)	23	8 – 45
AST (U/L)	40	15 – 37

On review of her medications, the patient had been started on 0.1% brimonidine tartrate eye drops, one drop twice a day to the right eye, for raised intraocular pressure one month before admission. The eye drops were stopped and her mental state improved markedly within one week. She became oriented to place and person. However, she was still lacking in motivation and complained of pervasive low mood, which was cautiously treated with a trial of the antidepressant vortioxetine. After a course of rehabilitation, she was discharged home with significantly improved cognition and was ambulatory with assistance.

## Discussion

Brimonidine normally undergoes hepatic metabolism via aldehyde oxidase to form oxo- and dioxo-brimonidine metabolites and is predominantly excreted in the kidney [2]. Brimonidine can cross the blood-brain barrier and cause central nervous system (CNS) depression via binding to α2-adrenergic receptors [1]. Delirium, psychosis, or even Charles-Bonnet syndrome has been reported in adults after ocular administration of brimonidine, and altered mental state has also been reported after topical use as a haemostatic agent in dermatologic surgery [1,3-5]. A variety of neuropsychiatric presentations, ranging from lethargy to coma, have been reported in neonates and children after administering brimonidine eye drops [6-8]. Depression has also been associated with increased α2-adrenoceptor activity in the brain in studies that used brimonidine as the detector ligand, and is a possible adverse effect of topical brimonidine; this may have been a contributing factor to the depressed mood and apathy observed in our patient [9]. Neurotoxicity is particularly common in elderly and paediatric age groups, and the effects are likely dose-dependent, extrapolating from early dose-response studies of brimonidine eye drops [2,10]. Reassuringly, as in our patient, complete neurological recovery was observed within days to weeks of drug cessation in most cases.

For lipophilic drugs, only around 10% of the active drug is absorbed into the eye via the cornea after topical administration of eye drops. Significant systemic drug absorption often occurs via the highly vascular nasal mucosa, after drainage through the nasolacrimal duct [11]. Importantly, such absorption bypasses hepatic first-pass metabolism and may increase the risk of systemic toxicities. Furthermore, for lipophilic drugs such as brimonidine, direct penetration into the CNS via the olfactory bulb after drainage into the nasal cavity has been demonstrated in animal studies [12]. In our case, neurotoxicity was evidently exacerbated by underlying advanced CKD with impaired drug excretion and systemic accumulation.

To reduce systemic absorption of topically administered eye drops, simple measures such as eyelid closure for up to five minutes and nasolacrimal duct occlusion may reduce systemic absorption by up to 67% and 65%, respectively [13,14]. Additionally, instillation of only one drop at a time is usually sufficient; whereas commercial eye drop dispensers often have a volume of 25-50 μL, the capacity of the conjunctival sac is only approximately 10 μL [15]. Unfortunately, dose adjustment of eye drops is logistically impossible even in patients with renal impairment. Brimonidine eye drops should therefore be used with caution in susceptible patients, including frail older adults and patients with impaired renal or liver function. Although alternative treatments may be available, ophthalmic preparations of beta-blockers and carbonic anhydrase inhibitors have also been associated with systemic side effects, including neuropsychiatric complications [16,17]. The smallest effective dose should be used, with vigilant monitoring for systemic toxicities.

There is no approved antidote for the treatment of brimonidine toxicity. Paediatric studies have described the use of activated charcoal after inadvertent oral ingestion of brimonidine eye drops, and also intravenous naloxone in established brimonidine toxicity, but no consistent benefit has been observed [8,18,19]. Conservative management with drug cessation and close monitoring was successful in most cases. Pertinent to our case, there are no data regarding whether haemodialysis may effectively remove the active compound from the circulation and facilitate recovery, especially in patients with severe acute or chronic renal impairment. Indeed, brimonidine does not have any safety data in advanced CKD. However, it is possible to predict from the known kinetics of brimonidine that it is probably poorly dialyzable; despite a relatively small molecular weight of 292.1 daltons, meaning that it can likely pass through the pores of a semi-permeable hemofilter membrane, brimonidine has a large volume of distribution of 10 L due to its lipid solubility [20]. Haemodialysis can only effectively remove drugs concentrated in the intravascular space. Significant redistribution of the active compound from the intracellular to the intravascular space is predicted to occur even in spite of removal during haemodialysis, by diffusion across cell membranes down a concentration gradient. An argument can be made for use of continuous renal replacement therapy, such as by CVVH, for continuous removal of redistributed brimonidine in oliguric patients, but no relevant reports were identified in the literature. Monitoring of drug levels will be helpful in case extracorporeal treatments are considered for reasons beyond standard indications for acute RRT. Given the above, it is plausible that the continuous removal of brimonidine by a prolonged session of CVVH given to our patient may have temporarily alleviated her symptoms, giving rise to the current, atypical presentation of delirium worsening after ICU discharge despite resolution of intercurrent sepsis. These symptoms were minimally improved even after the institution of regular intermittent haemodialysis, probably due to continuous administration of the offending eye drops and the inefficiency of intermittent RRT, with significant post-dialysis drug rebound with redistribution from other fluid compartments after each session of haemodialysis.

## Conclusions

We have reported on a case of drug-induced encephalopathy in a patient with underlying advanced CKD, after administration of brimonidine eye drops. This case illustrates the need to pay close attention to the drug history, including topical medications, especially in patients with risk factors for systemic toxicity including abnormal end-organ function. The role of antidotes and extracorporeal treatments for various drug-induced encephalopathies, including those due to brimonidine, remains to be defined. Ultimately, a high index of suspicion and early cessation of offending medications remain the cornerstones of treatment, in order to prevent life-threatening complications and obviate the need for expensive and invasive investigations in the workup for delirium.
